# Bibliometric Analysis of Global Scientific Production on COVID-19 and Vaccines

**DOI:** 10.3390/ijerph20064796

**Published:** 2023-03-08

**Authors:** Antonio Rosa de Sousa Neto, Ana Raquel Batista de Carvalho, Márcia Daiane Ferreira da Silva, Marly Marques Rêgo Neta, Inara Viviane de Oliveira Sena, Rosângela Nunes Almeida, Francidalma Soares Sousa Carvalho Filha, Laianny Luize Lima e Silva, Girlene Ribeiro da Costa, Ivana Mayra da Silva Lira, Denise Maria Meneses Cury Portela, Amanda Tauana Oliveira e Silva, Cristiane Borges de Moura Rabêlo, Andreia Rodrigues Moura da Costa Valle, Maria Eliete Batista Moura, Daniela Reis Joaquim de Freitas

**Affiliations:** 1 Nursing Department, Federal University of Piaui, Teresina 64049-550, Brazil; 2 Department of Medicine, Centro Universitário Uninovafapi, Teresina 64073-505, Brazil; 3 Health Sciences Center, Federal University of Piaui, Teresina 64001-020, Brazil; 4 Department of Parasitology and Microbiology, Federal University of Piaui, Teresina 64049-550, Brazil

**Keywords:** COVID-19 vaccines, vaccine, bibliometric indicators

## Abstract

This bibliometric analysis aims to analyze the global scientific production of COVID-19 and vaccines. First, a search for scientific articles was performed using the advanced query in the Web of Science™ database, more precisely in its core collection, on 18 February 2023. Data from 7754 articles were analyzed using the Bibliometrix R package and the Biblioshiny application. The evaluated articles were published mainly in 2022 (60%). The scientific journals that published the most about COVID-19 and vaccines were “Vaccines”, “Vaccine” and “Human Vaccines & Immunotherapeutics”. The University of Oxford was the most productive institution, with the authors of the articles mainly originating from the United States, China and the United Kingdom. The United States, despite having carried out the most significant number of collaborations, published mainly with local researchers. The 15 most cited articles and the KeyWords Plus™ evidenced the focus of the published articles on the safety and efficacy of vaccines against COVID-19, as well as on the evaluation of vaccine acceptance, more specifically on vaccine hesitancy. Research funding came primarily from US government agencies.

## 1. Introduction

Coronaviruses (CoVs) were discovered around 1960 [1]. Such viruses can cause disease in humans and animals, causing mild to severe respiratory manifestations and even death. Among the seven CoV types of CoVs that cause human disease, the zoonotic coronaviruses SARS-CoV and MERS-CoV stand out as highly pathogenic, causing Severe Acute Respiratory Syndrome (SARS) [2]. In this context, the variation of SARS-CoV, SARS-CoV-2, deserves attention as the agent causative of the 2019 Coronavirus disease (COVID-19) that triggered the present pandemic [3].

At the time of the discovery of SARS-CoV-2, the World Health Organization (WHO) recommended adopting different measures to prevent COVID-19, such as isolation, quarantine, social distancing and the use of masks and hand sanitation (washing with soap and water or using gel alcohol). Currently, with the creation of vaccines against COVID-19, global incentives have focused mainly on vaccinating the entire population [4].

Vaccines are a great form of prevention and control, as they were responsible for eradicating smallpox, a disease that caused the death of about 400 million people in the 20th century alone [5].

In the context of COVID-19, due to the emergency and lethality of the virus, to minimize mortality and the occurrence of severe cases, the time for designing and developing a vaccine was reduced to approximately one year [6]. The first vaccine to receive an emergency use validation by the WHO Strategic Advisory Group of Experts on Immunization (SAGE) for efficacy against COVID-19 was that of Pfizer/BioNTech on 5 January 2021 [7].

Bibliometric analysis or bibliometrics has gained notoriety due to high scientific production [8]. This analysis is an effective method used to quantitatively measure and portray scientific research, which can elucidate critical data, filter important work by estimating its impact and discover the underlying structure of a field [9].

In the scenario of the COVID-19 topic, this type of analysis can indicate possible directions to be explored by scientists, promote cooperation with other researchers and guide emerging researchers toward specialties that still need to be developed. Furthermore, it may enable the development and use of new technologies in this field [10].

In order to understand and give visibility to works related to the subject, this bibliometric analysis aims to analyze the global scientific production of COVID-19 and vaccines.

## 2. Materials and Methods

The present study is descriptive, bibliometric with a quantitative approach and guided by the five steps recommended in bibliometric research: research design, a compilation of bibliometric data, data analysis, data visualization and interpretation of results [9]. Notably, this type of analysis allows researchers to investigate more data than systematic literature reviews, maintaining high rigor, scientific soundness, transparency and replicability [11,12].

First, a search for scientific articles was performed using the advanced query in the Web of Science™ (WoS) database, more precisely in its core collection, on 18 February 2023. Web of Science™ is among the most reliable and comprehensive databases for bibliometric studies, allowing the tracking of nearly 1.9 billion references cited from over 171 million records [13].

To formulate the search strategy, descriptors from the Medical Subject Headings (MeSH), Boolean operators and wildcard characters were used. The strategy created and used was the following: “TI = ((COVID OR “SARS CoV 2” OR “Coronavirus Disease 2019” OR “Coronavirus Disease 19” OR “2019 nCoV”) AND (vaccine *))”.

To specify the subject, increase precision and reduce false-positive results, the present study considered only the titles of the articles. The literature has already reported that specific searches by titles increase retrieval and specificity, generating a minimal loss of sensitivity compared to a search that includes all fields [14,15].

Only original articles published until 31 December 2022 and published in English were included. Articles outside the scope of the research, review articles, opinion articles, reflection articles, editorials, case studies and articles with a publication date of 2023 were excluded.

The first search in the database resulted in 14,973 articles. After filtering and applying the criteria, 7754 articles remained. These had all available information downloaded in text file format for analysis. Bibliometric research also describes these data as “metadata” [9,16].

**Figure 1:**
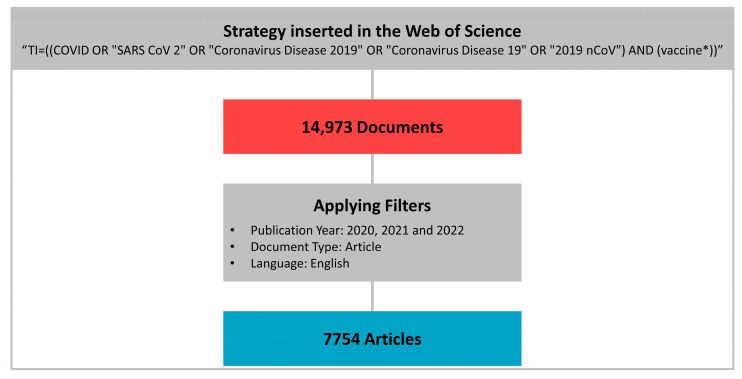
Selection of articles for analysis.

The metadata were imported into the RStudio Desktop Software (v.2022.12.0 + 353)—linked to the R Software (v.4.2.1)—and converted into an R data frame. The Bibliometrix R package (http://www.bibliometrix.org (accessed on 1 January 2023)) and the Biblioshiny application, which provides a web interface, were used for analysis. Bibliometrix is an open-source tool to perform a comprehensive scientific mapping analysis of scientific literature, programmed in R to be flexible and facilitate integration with other statistical and graphics packages [16]. Figure 1 summarizes how the articles were selected for inclusion in this research.

The analysis made it possible to visualize the production of articles according to the year of publication, the scientific journals that published the most, the institutions most involved in the research and the countries according to the authors’ affiliations. Likewise, it was possible to identify the collaborations, the most cited articles, the conceptual structure according to KeyWords Plus™ and the funding sources.

## 3. Results

### 3.1. Publication Year

**Figure 2:**
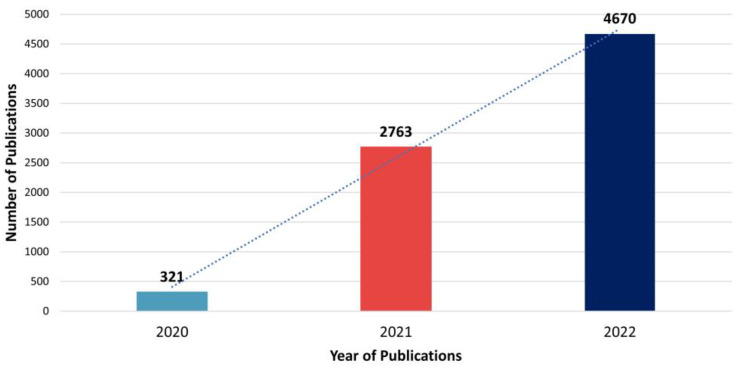
Annual published articles on the subject according to WoS data.

Of the 7754 articles evaluated, most were published in 2022 (60%), followed by 2021 (36%) and 2020 (4%). That is, there was an exorbitant growth rate of 761% from 2020 to 2021 and about 69% from 2021 to 2022. Figure 2 shows the annual publication of articles on the subject.

### 3.2. Scientific Journals

The articles in the dataset were published in 1815 scientific journals, of which only 694 (38%) published more than three articles and 294 (16%) more than two articles, with an average of 4.3 articles per journal. The 15 most productive journals had approximately 30% of the total articles retrieved and belonged to different research areas—such as immunology, medicine and public health—with almost all in the health area according to the WoS categories (Table 1).

### 3.3. Affiliate Institutions

Table 2 presents the 15 institutions most involved in research on the subject. When assessing co-occurrence, Bibliometrix identified 9850 institutions in the authors’ affiliations. Notably, the same institution may be present more than once in each article. According to the findings, 5909 (60%) institutions appeared only once. The University of Oxford was the affiliate institution that was most present in the surveys, obtaining a frequency of 368 appearances.

### 3.4. Countries and Collaborations

**Figure 3:**
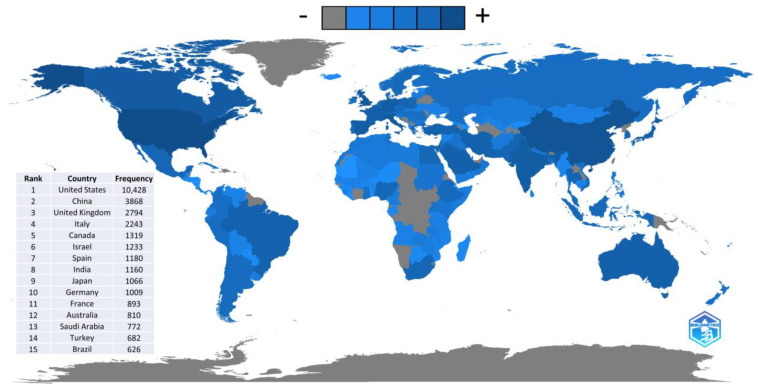
Scientific production by country according to authors’ affiliation.

The articles were produced by 43,344 authors from 138 countries, as recognized by Bibliometrix. The geographic distribution of the articles is shown in Figure 3. The figure is based on the co-occurrence of the countries according to the authors’ affiliations, which explains the frequency found to be greater than the number of articles evaluated. In the figure, gray indicates the absence of local authors, while shades of blue—from lighter to darker—indicate an increase in local authors.

When considering the 15 most productive countries, it is possible to observe that these belong mainly to the American, Asian and European continents. In this way, the articles were produced mainly by researchers from the United States (USA), who were the top producers and were present 10,428 times. Other countries that stood out when considering the affiliation of their researchers were: China (*n* = 3868), the United Kingdom (*n* = 2794), Italy (*n* = 2243), Canada (*n* = 1319), Israel (*n* = 1233), Spain (*n* = 1180), India (*n* = 1160), Japan (*n* = 1066) and Germany (1009).

However, when evaluating the collaboration rate—defined as the ratio between the number of multi-country collaborations and the total number of articles attributed considering the affiliation of the corresponding author (Table 3)—among the top 15 countries per publication, it is evident that the United States, despite having carried out the highest number of collaborations, published mainly with local researchers. On the other hand, countries such as Germany and the United Kingdom have a high rate of multi-country publications, getting 42% and 40%, respectively.

**Figure 4:**
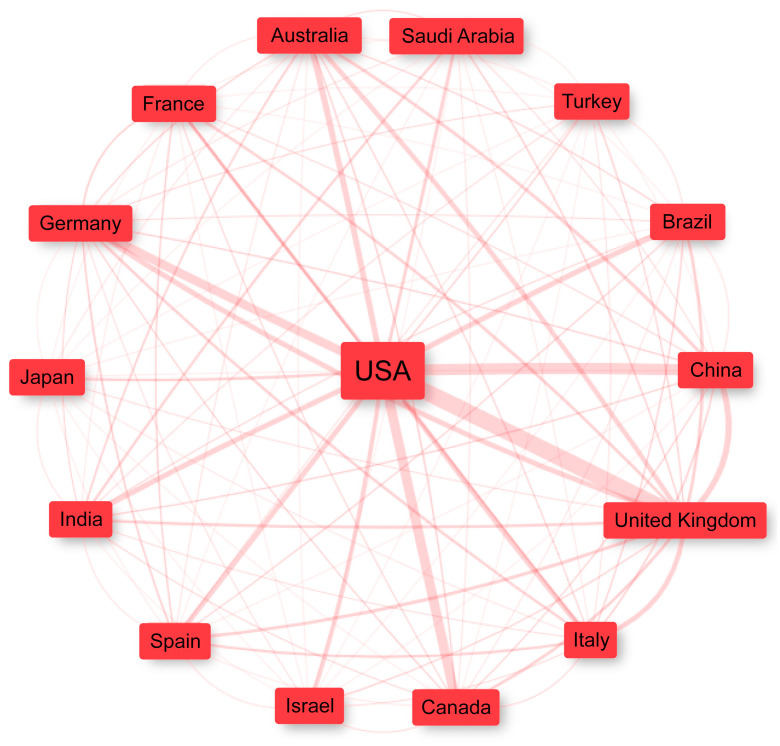
Collaboration network of the main countries.

Regarding the collaboration network, when considering the co-occurrence of the 50 main countries in the authors’ affiliations, adopting the Leiden clustering algorithm [17], only one cluster was generated, demonstrating the high occurrence of collaboration. For better visualization, Figure 4 (star network diagram) has been limited to the top 15 countries. Notably, the box’s size is proportional to the number of times the country appears, with the width of the link becoming more robust as the number of publications together increases. The countries that most collaborated with different countries were the United States, the United Kingdom, China and Italy (with the United States collaborating mainly with the United Kingdom, China, Canada and Germany).

### 3.5. Most Cited Articles

The 7754 articles were cited 158,890 times, with an average of 20.49 citations per item. Citations of the top 15 articles ranged from 6729 to 779 (Table 4). The most cited articles are from five different journals and were published in 2020 and 2021.

### 3.6. Conceptual Structure

**Figure 5:**
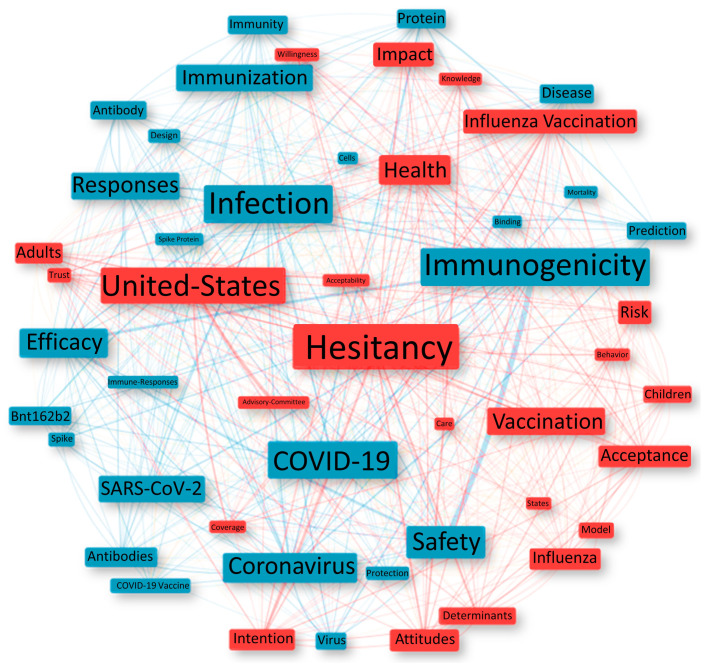
The conceptual structure according to KeyWords Plus™.

Keywords can summarize the focus of articles and determine search trends [33]. Therefore, to evaluate the conceptual structure, the most frequent KeyWords Plus™ (index terms automatically generated from the titles of articles) were selected, as already performed in other studies [34,35,36]. The Leiden clustering algorithm [17] (sphere network scheme) was adopted again, with the box size proportional to the number of times the term appears. The analysis generated two clusters, with terms demonstrating the focus of the research on evaluating the ability of vaccines against COVID-19 to generate immunogenicity (26 terms in blue) and on evaluating the acceptance of vaccines, more specifically on vaccine hesitancy (24 terms in red) as shown in Figure 5.

### 3.7. Financing Agencies

According to data made available by WoS, eight of the top fifteen funding sources are from the United States. Another important finding is that resources come mainly from government agencies, as shown in Table 5. Notably, for the composition of the table, only data from surveys that reported funding were considered; that is, only 50.3 % of the total of 7754.

## 4. Discussion

This bibliometric analysis of research related to COVID-19 and Vaccines covered the original production from 2020 to December 2022. The growth of publications from 2020 to 2021 was evident, which may have occurred due to the creation of vaccines; the first vaccine was approved in January 2021 by the WHO [7].

According to data from 2 December 2022, the WHO has already approved 11 vaccines for emergency use. Two vaccines are of the protein subunit type, two of ribonucleic acid (RNA), four of non-replicating viral vectors and three of the inactivated viruses [37].

The researchers’ choice to publish in journals in the immunology category was evident when evaluating Table 1. Immunology studies the immune responses and cellular and molecular events that occur when the organism identifies microorganisms and other foreign macromolecules [38]. Vaccines and immunology are connected, as vaccines are antigenic preparations that induce the immune response of individuals and can prevent the onset of diseases or attenuate clinical manifestations [39].

The scientific journal that most published articles related to the topic addressed was “Vaccines” (Journal Citation Reports™ 2021: 4961). This discovery correlates with the fact that the journal is focused on laboratory and clinical research on vaccines, also addressing their use and immunization, which influenced it to be the authors’ first choice. One of their significant studies, conducted before vaccines were created, reported the high acceptance of COVID-19 vaccination among the Chinese population during the current pandemic [40].

The second most published journal was “Vaccine” (Journal Citation Re-ports™ 2021: 4169). As per its scope, it is a relevant journal for vaccinology, covering original articles in basic and clinical research, vaccine manufacturing, history, public policy, behavioral science and ethics, social sciences, safety and other related areas. One of its most cited studies was a survey conducted before the creation of vaccines with adults in the United States, which identified the acceptability of vaccination in 69% of respondents [41].

The third journal was “Human Vaccines & Immunotherapeutics” (Journal Citation Reports™: 4526). This journal is dedicated to the publication of international research in vaccinology and immunotherapy and the exploration of new and experimental vaccines. One of its most relevant studies was carried out in Malaysia and had as its main result the intention of the interviewees to receive the vaccine against COVID-19 [42].

Thus, when considering the three leading journals based on their publications, it was evident that the researchers focused on publishing their work in journals with the specific scope of the subject. Likewise, it was evident that the three journals began to publish on the subject even in the absence of vaccines, first addressing the acceptability of vaccines against COVID-19 in different countries and contexts.

When evaluating the institutions that produced the articles, it is observed that nine of the fifteen institutions are located in the US, demonstrating the efforts of that country, which carried out collaborations with pharmacists, biotechnologists and academics, aiming to capitalize on several decades of progress in new vaccine platforms, viral immunology, structural biology and protein engineering research, along with clinical trial operations expertise to enable the rapid development, evaluation, manufacture and deployment of successful vaccines [43].

On the other hand, the most productive institution was the University of Oxford, located in the United Kingdom. Researchers from that institution started to publish in early 2020 on the subject when discussing the record time for the first human trials of the vaccine against COVID-19 and the assessment of the risk of increasing the disease with vaccines [44,45]. In addition, it obtained a successful partnership with the pharmaceutical company AstraZeneca, which culminated in the development of the Vaxzevria™ vaccine [46,47]. Consequently, this institution also carried out studies evaluating the efficacy of vaccines against SARS-CoV-2 variants [22,48,49].

The evaluation of the countries’ production demonstrated the global effort in developing vaccines against COVID-19 since about 71% of the countries participated in research on the subject if one considers the count of 193 countries, according to the United Nations [50]. In this context, the participation of researchers from the United States was significant. It is of note that an article from March 2021 agreed with the present research when describing that the United States, China and the United Kingdom already led both in the number of candidate vaccines against COVID-19 and in the percentage of publications in journals [51].

Therefore, it is evident that although the top 15 institutions belong to only five countries, several collaborations were carried out, allowing the participation of countries worldwide. In addition, it is essential to emphasize that although the main collaborations were carried out between countries with high levels of development, it was precisely due to the sharing of knowledge and techniques between the countries that the creation of vaccines was made possible, with the magazine Science pointing this out as the most significant advance of 2020 [52,53].

The 15 most cited articles addressed two main themes: the evaluation of vaccine efficacy and safety in 13 articles [18,19,20,21,22,23,24,25,27,28,29,31,32] and vaccine hesitancy in 2 [26,30]. Another finding is that such a ranking has no article published in 2022.

It is essential to highlight that the BNT162b2 vaccine, by Pfizer/BioNTech, called Comirnaty™, was addressed in five of the thirteen most cited articles [54]. In one article, BNT162b2 demonstrated a lower incidence and severity of systemic reactions than another candidate (BNT162b1), ensuring the continuity of its tests [25]. In other studies, BNT162b2 continued to show protection against COVID-19, also succeeding against the Delta variant (B.1.617.2) [18,22,23,32]. Tests with the other candidate (BNT162b1) by Pfizer/BioNTech were addressed in two of the most cited studies [25,29].

The second most discussed vaccine, when considering Table 4, was Vaxzevria™, being present in three articles that included its initial tests, its acceptable safety, tests carried out in different countries on different continents (Brazil, South Africa and the United Kingdom) and demonstrations of its efficacy against the Delta variant [20,22,24]. Consequently, the Spikevax™ [19,21,55], Jcovden™ [27,56] and Sputnik V™ [28,57] vaccines appear less frequently, where the articles mainly addressed safety and efficacy.

Despite not being present in Table 1, the scientific journal “The New England Journal of Medicine”, which has been published continuously for more than 200 years (Journal Citation Reports™ 2021: 176,082), appeared eight times in the list of the most cited articles, with their articles covering the Comirnaty™, Spikevax™ and Jcovden™ vaccines [18,19,21,22,23,25,27,31]. The journal “The Lancet” (Journal Citation Reports™ 2021: 202,731) appeared three times, publishing about the Vaxzevria™ and Sputnik V™ vaccines [20,24,28].

As previously reported, two of the fifteen articles addressed vaccine hesitancy [26,30], the popular term since 2015, when the WHO Strategic Advisory Group of Experts on Immunization defined vaccine hesitancy as the delay in accepting or refusing vaccination despite the availability of vaccination services, which can vary in form and intensity, depending on the moment, the place of occurrence or the vaccine involved [58,59,60].

In the present research, it was evident, as shown in Figure 5, that researchers have been working effectively on vaccine hesitancy since before the creation of vaccines against COVID-19 [61,62]. This topic is significant, since the sharing of false news mainly by “anti-vaxxers”, as has occurred in an exacerbated way since the beginning of the pandemic, can trigger vaccine hesitancy and, consequently, result in a decrease in the behavioral intention of population vaccination [63,64,65].

Thus, the red cluster demonstrates that several studies on vaccine hesitancy were published worldwide, but mainly contemplating the United States population [41,61,66,67,68,69]. The blue cluster addresses immunogenicity, defined as the ability of a foreign substance, such as an antigen, to provoke an immune response; that is, efficacy, which, together with safety assessment, make up essential and necessary phases for the approval of new vaccines [6,70].

Consequently, although no 2022 article appeared among the most cited, its three most cited articles followed the others, addressing the efficacy of vaccines against variants and vaccine hesitancy. Namely, the most cited in 2022 sought to estimate the efficacy of three vaccines against symptomatic disease caused by the Omicron and Delta variants (B.1.617.2) in United Kingdom [49]. The second article addressed the effects of boosters with mRNA-based vaccines against the Omicron variant (BA.1/B.1.1.529) and the third evaluated vaccine hesitancy in the United Kingdom [71,72].

Research funding came mainly from the United States through its agencies. It is worth noting that, in addition to the identified sources, mainly from government agencies and private and philanthropic entities, many researchers funded their research, again emphasizing the joint effort to produce evidence and combat the COVID-19 pandemic [73].

Finally, the main current challenge is to obtain herd immunity and “vaccine equity” among countries, bearing in mind that the challenges of vaccines against COVID-19 include three dimensions, ranging from development and dissemination to the distribution of vaccines [74,75]. Vaccine distribution is still a problem in countries with low human development, which still lack vaccines and strategies to exceed the target of 70% of the population vaccinated [76,77].

Therefore, studies that contemplate the efficacy and safety of vaccines in the long term must continue to be carried out, as well as those related to vaccine hesitancy, in different contexts, aiming to formulate effective strategies to increase vaccination rates aiming at the end of the COVID-19 pandemic.

## 5. Limitations

The limitations are the use of only one database and the delimitation of the research to the titles, which can lead to minimal losses, as stated in materials and methods. This study included articles published until 31 December 2022, not including publications after that date, and considering that the theme is still recent, a new study will be necessary posteriorly.

## 6. Conclusions

The 7754 evaluated articles were published mainly in 2022 (60%). The scientific journals that published the most about COVID-19 and vaccines were “Vaccines”, “Vaccine” and “Human Vaccines & Immunotherapeutics”. The University of Oxford was the most productive institution, with the authors of the articles mainly originating from the United States, China and the United Kingdom. The United States, despite having carried out the most significant number of collaborations, published mainly with local researchers. The 15 most cited articles and KeyWords Plus™ evidenced the focus of the published articles on the safety and efficacy of vaccines against COVID-19, as well as on the evaluation of vaccine acceptance, more specifically on vaccine hesitancy. Research funding came primarily from US government agencies.

## Figures and Tables

**Table 1:** Ranking of scientific journals that most published on COVID-19 and vaccines.

Rank	Journal	WoS Categories	Articles
1	Vaccines	Medicine, Research and Experimental/Immunology	788
2	Vaccines	Medicine, Research and Experimental/Immunology	288
3	Hum. Vaccin. Immunother.	Biotechnology and Applied Microbiology/Immunology	232
4	Front. Immunol.	Immunology	168
5	PloS One	Multidisciplinary Sciences	144
6	Int. J. Environ. Res. Public Health	Public, Environmental and Occupational Health	127
7	Front. Public Health	Public, Environmental and Occupational Health	121
8	Cureus J. Med. Sci.	Medicine, General and Internal	94
9	Clin. Infect. Dis.	Microbiology/Infectious Diseases/Immunology	82
10	Sci. Rep.	Multidisciplinary Sciences	77
11	Nat. Commun.	Multidisciplinary Sciences	68
12	J. Med. Virol.	Virology	57
13	Morb. Mortal. Wkly. Rep.	Public, Environmental and Occupational Health	57
14	Int. J. Infect. Dis.	Infectious Diseases	56
15	NPJ Vaccines	Medicine, Research and Experimental/Immunology	55

**Table 2:** Ranking of affiliate institutions that most published on COVID-19 and vaccines.

Rank	Affiliate Institutions	Country	Frequency
1	University of Oxford	United Kingdom	368
2	The University of Hong Kong	China	204
3	Harvard Medical School	United States	199
4	Tel Aviv University	Israel	191
5	University of Pennsylvania	United States	190
6	University of Washington	United States	190
7	University of Toronto	Canada	189
8	Stanford University	United States	166
9	Emory University	United States	157
10	University of Michigan	United States	155
11	Johns Hopkins University	United States	136
12	Fudan University	China	135
13	Washington University	United States	135
14	University of California San Francisco	United States	134
15	Imperial College London	United Kingdom	129

**Table 3:** Production and collaborations of countries according to corresponding author.

Rank	Country	Articles	SCP	MCP	MCP_Rate
1	United States	1997	1631	366	18%
2	China	812	626	186	23%
3	Italy	465	388	77	17%
4	United Kingdom	400	239	161	40%
5	India	276	213	63	23%
6	Israel	225	180	45	20%
7	Japan	223	202	21	9%
8	Canada	204	133	71	35%
9	Turkey	187	180	7	4%
10	Spain	186	151	35	19%
11	Saudi Arabia	173	108	65	38%
12	Germany	163	94	69	42%
13	France	162	114	48	30%
14	Australia	155	106	49	32%
15	Korea	126	104	22	18%

Legend: Country = Country of the corresponding author’s affiliation; Articles = Number of articles per country of corresponding author’s affiliation; SCP = Single Country Publication; MCP = Multi Country Publication; MCP_rate = Multi-country publication rate.

**Table 4:** Ranking of most cited published articles on COVID-19 and vaccines.

Rank	Author (Year), Journal	Title	Total Citations (TC)
1 [18]	Polack, F.P. et al. (2020), N. Engl. J. Med.	Safety and Efficacy of the BNT162b2 mRNA COVID-19 Vaccine	6729
2 [19]	Baden, L.R. et al. (2021), N. Engl. J. Med.	Efficacy and Safety of the mRNA-1273 SARS-CoV-2 Vaccine	4486
3 [20]	Voysey, M. et al. (2021), Lancet	Safety and efficacy of the ChAdOx1 nCoV-19 vaccine (AZD1222) against SARS-CoV-2: an interim analysis of four randomised controlled trials in Brazil, South Africa and the UK	2259
4 [21]	Jackson, L.A. et al. (2020), N. Engl. J. Med.	An mRNA Vaccine against SARS-CoV-2—Preliminary Report	1715
5 [22]	Bernal, J.L. et al. (2021), N. Engl. J. Med.	Effectiveness of COVID-19 Vaccines against the B.1.617.2 (Delta) Variant	1527
6 [23]	Dagan, N. et al. (2021), N. Engl. J. Med.	BNT162b2 mRNA COVID-19 Vaccine in a Nationwide Mass Vaccination Setting	1312
7 [24]	Folegatti, P.M. et al. (2020), Lancet	Safety and immunogenicity of the ChAdOx1 nCoV-19 vaccine against SARS-CoV-2: a preliminary report of a phase 1/2, single-blind, randomised controlled trial	1304
8 [25]	Walsh, E.E. et al. (2020), N. Engl. J. Med.	Safety and Immunogenicity of Two RNA-Based COVID-19 Vaccine Candidates	1299
9 [26]	Lazarus, J.V. et al. (2021), Nat. Med.	A global survey of potential acceptance of a COVID-19 vaccine	1248
10 [27]	Sadoff, J. et al. (2021), N. Engl. J. Med.	Safety and Efficacy of Single-Dose Ad26.COV2.S Vaccine against COVID-19	1188
11 [28]	Lugunov, D.Y. et al. (2021), Lancet	Safety and efficacy of an rAd26 and rAd5 vector-based heterologous prime-boost COVID-19 vaccine: an interim analysis of a randomised controlled phase 3 trial in Russia	838
12 [29]	Mulligan, M.J. et al. (2020), Nature	Phase I/II study of COVID-19 RNA vaccine BNT162b1 in adults	827
13 [30]	Dror, A.A. et al. (2020), Eur. J. Epidemiol.	Vaccine hesitancy: the next challenge in the fight against COVID-19	814
14 [31]	Anderson, E.J. et al. (2020), N. Engl. J. Med.	Safety and Immunogenicity of SARS-CoV-2 mRNA-1273 Vaccine in Older Adults	790
15 [32]	Haas, E.J. et al. (2021), Lancet	Impact and effectiveness of mRNA BNT162b2 vaccine against SARS-CoV-2 infections and COVID-19 cases, hospitalizations and deaths following a nationwide vaccination campaign in Israel: an observational study using national surveillance data	779

**Table 5:** Ranking of funding sources for research on COVID-19 and vaccines.

Rank	Funding Source	Country	Type	Frequency
1	U.S. Department of Health Human Services (HHS)	United States	Government Department	670
2	National Institutes of Health (NIH)	United States	Government Agency	604
3	National Natural Science Foundation of China (NSFC)	China	Government Agency	238
4	European Commission (EC)	Belgium	Parliament	174
5	National Institute of Allergy and Infectious Diseases (NIAID)	United States	Government Agency	164
6	UK Research and Innovation (UKRI)	United Kingdom	Government Agency	147
7	Medical Research Council (MRC)	United Kingdom	Government Agency	99
8	Centers For Disease Control Prevention (CDC)	United States	Information Service	83
9	Wellcome Trust	United Kingdom	Philanthropic Institution	83
10	Bill and Melinda Gates Foundation (BMGF)	United States	Philanthropic Institution	78
11	National Science Foundation (NSF)	United States	Government Agency	77
12	National Institute for Health and Care Research (NIHR)	United Kingdom	Government Agency	74
13	Canadian Institutes of Health Research (CIHR)	Canada	Government Agency	64
14	National Cancer Institute (NCI)	United States	Government Agency	59
15	Pfizer	United States	Company	48
